# Vesicles from Amphiphilic Dumbbells and Janus Dendrimers: Bioinspired Self-Assembled Structures for Biomedical Applications

**DOI:** 10.3390/polym9070280

**Published:** 2017-07-12

**Authors:** Soraya Taabache, Annabelle Bertin

**Affiliations:** 1 Federal Institute for Materials Research and Testing (BAM), Department 6.0, D-12205 Berlin, Germany; soraya.taabache@bam.de; 2 Fraunhofer ICT-IMM, D-55129 Mainz, Germany; 3 Institute of Chemistry and Biochemistry, Freie Universität Berlin, D-14195 Berlin, Germany

**Keywords:** Janus dendrimers, amphiphilic dumbbells, self-assembled structures, vesicles, dendrimersomes, drug delivery systems, theranostics, artificial cells, lectin recognition, self-sorting

## Abstract

The current review focuses on vesicles obtained from the self-assembly of two types of dendritic macromolecules, namely amphiphilic Janus dendrimers (forming dendrimersomes) and amphiphilic dumbbells. In the first part, we will present some synthetic strategies and the various building blocks that can be used to obtain dendritic-based macromolecules, thereby showing their structural versatility. We put our focus on amphiphilic Janus dendrimers and amphiphilic dumbbells that form vesicles in water but we also encompass vesicles formed thereof in organic solvents. The second part of this review deals with the production methods of these vesicles at the nanoscale but also at the microscale. Furthermore, the influence of various parameters (intrinsic to the amphiphilic JD and extrinsic—from the environment) on the type of vesicle formed will be discussed. In the third part, we will review the numerous biomedical applications of these vesicles of nano- or micron-size.

## 1. Introduction

Vesicles self-assembled in aqueous environment from natural and synthetic phospholipids (liposomes) [1,2], amphiphilic block copolymers (polymersomes) [3,4], and more recently amphiphilic Janus dendrimers (dendrimersomes) [5] as hollow soft structures in the nano- or micron-size regime have attracted much interest. The sustained interest in vesicular assemblies arises from their unique structure, as vesicles can be configured into biomimetic nanocapsules and encapsulate water-soluble cargo inside their inner aqueous cavity as well as water-insoluble payload inside their membrane [6]. This enables various applications of vesicles as carriers in drug, protein or gene delivery [7,8], diagnostic imaging [9] or theranostics [10]. Moreover, as vesicles can mimic primitive and contemporary biological membranes, their potential as artificial cells has also arisen [11,12].

Liposomes, first discovered by Bangham et al. in 1961, are vesicles self-assembled from natural phospholipids [13]. The bilayer thickness is similar to the one of living cells (~4 nm) as liposomes are made from the same building blocks. Liposomes are semi-permeable vesicles that are usually polydisperse, unstable, with poor mechanical properties and require multiple fractionations (most conveniently by extrusion) in order to generate monodisperse vesicles of specific size [14]. Liposomes made their successful entry into the market in 1995 with the development of PEGylated liposomal formulations, such as Doxil^®^ [15]. These “stealth” liposomes showed an improved stability, bioavailability and limited toxicity, thereby overcoming the limitations of traditional liposomes [16]. Unfortunately, the number of building blocks, i.e., lipids, is limited.

Unlike liposomes, polymersomes first reported by the groups of Discher and Hammer in 1999 [3] present an often relatively impermeable membrane with a membrane thickness larger than typical cell membranes (controlled by the molecular weight of the building blocks) [17,18]. Moreover, their building blocks, i.e., amphiphilic block copolymers, present an inherent polydispersity. From this point of view, polymersomes seem less suitable as bioinspired vesicles. Nonetheless, they present also advantages as they are usually mechanically more stable than liposomes and their chemical structure can be varied based on a wide range of monomers that are commercially available allowing also the preparation of stimuli-responsive structures [19,20].

Dendrimersomes (DSs) are vesicles formed by the self-assembly of amphiphilic Janus dendrimers [21]. Janus dendrimers, which are dendrimers constituted of two dendrimeric wedges with distinct properties, are not the focus of this review but are treated with great details in the excellent reviews of the groups of Caminade and Majoral [22] or Govender [23]. DSs combine the advantages from liposomes and polymersomes: they are known to be stable over a longer period of time like polymeric vesicles and less permeable than lipid vesicles. While polymeric building blocks are polydisperse in molecular weight resulting in a broad size distribution of polymersomes, amphiphilic Janus dendrimers (JDs) have a well-defined molecular weight [22,24]. Moreover, because of their membrane thickness similar to that of cells, dendrimersomes can also be considered as a biomimetic model of biological membranes or as artificial cells. Finally, JDs can be synthesized using the full range of building blocks and syntheses that organic chemistry offers [24,25,26], which may be the greatest advantage of dendrimersomes over liposomes and polymersomes.

Amphiphilic dumbbells that, similar to JDs, belong to the dendritic family, also have a well-defined molecular weight but are constituted of two flexible dendrons coupled on each side of a rigid hydrophobic core. Even though they are far less studied than JDs, they also show a propensity to form vesicles in aqueous media.

The current review focuses on vesicles obtained from the self-assembly of two types of dendritic macromolecules, namely amphiphilic Janus dendrimers (forming dendrimersomes) or amphiphilic dumbbells. We will present in a first part the synthetic strategies and the repertoire of building blocks (hydrophilic dendron, hydrophobic dendron, core) that can be used to obtain dendritic-based macromolecules. We put our focus on amphiphilic Janus dendrimers and amphiphilic dumbbells that form vesicles in water, but we also encompass vesicles formed thereof in organic solvents. In the second part of this review, we will discuss the different preparation methods of these vesicles at the nanoscale and microscale. Moreover, the influence of various parameters (solution concentration, solvent, etc.) on the vesicle properties, such as the size, lamellarity, or biocompatibility will be considered. In the third part, we will focus on the biomedical applications of these vesicles. At the nanoscale, they can be used as nanocarriers for drugs, imaging agents or a combination of both (nanotheranostics). Furthermore, they can be employed as tools for understanding the role of membrane proteins or lectin recognition on cells. At the microscale these vesicles can be used as artificial cells being able to integrate membrane proteins and showing the capacity of self-sorting, which is found in Nature. Finally, throughout this review, we will try to highlight the potential of vesicles from amphiphilic dumbbells and Janus dendrimers as alternative to liposomes and polymersomes.

## 2. Amphiphiles of the Dendritic Family

**Figure 1:**
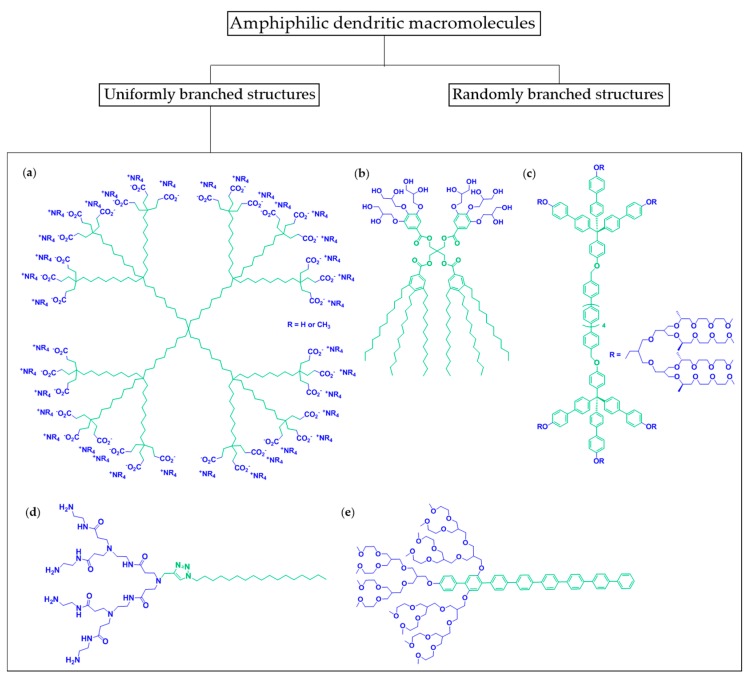
Examples for each sub-group of uniformly branched dendritic macromolecules: (**a**) amphiphilic dendrimer [28]; (**b**) amphiphilic JD [21]; (**c**) amphiphilic dumbbell [34]; (**d**,**e**) dendritic amphiphiles with flexible hydrophobic chain [57] and rigid hydrophobic segment [46], respectively.

The dendritic family comprises macromolecules with randomly branched structures such as hyperbranched polymers, dendrigrafts or dendritic-linear hybrids as well as macromolecules with uniformly branched structures [27]. The name “dendrimer” originates from the Greek word δέντρο (dendro), which means “tree”. Amphiphiles that belong to the second category, namely macromolecules with uniformly branched structures, are characterized by a well-defined architecture and molecular weight. They comprise amphiphilic dendrimers (Figure 1a), amphiphilic JDs (Figure 1b), amphiphilic dumbbells (Figure 1c), and dendritic amphiphiles (Figure 1d with flexible hydrophobic chain and Figure 1e with rigid hydrophobic segment). These compounds can be classified according to the number of repeated branching cycles that are performed during their synthesis, also called generation (G0, G1, etc.).

Amphiphilic dendrimers have a core/shell structure and behave as “unimolecular micelles” in a polar solvent when they are constituted of an apolar core and a polar shell, i.e., in water a hydrophobic interior and hydrophilic periphery [28,29,30]. Alternatively, amphiphilic dendrimers can possess both hydrophobic and hydrophilic dendritic parts coupled to a core [31].

Amphiphilic JDs are composed of a hydrophilic dendron and a hydrophobic dendron linked to the same core [21]. A dendron usually contains a single chemically addressable group called the focal point. A distinction between amphiphilic JDs and amphiphilic dendrimers is sometimes difficult as the boundaries are blurred [31]. However, a difference between these two types is justified as the shape of amphiphilic JDs is generally not globular, while the shape of amphiphilic dendrimers is. Additionally, it is to be noted that the term “amphiphilic dendrimer” is widely used in the literature in relation with non-globular amphiphiles [32,33].

Amphiphilic dumbbells are constituted of two flexible dendrons coupled on each side of a rigid hydrophobic core [34,35,36,37,38,39,40]. Amphiphiles with a non-dendritic hydrophobic structure (for instance, one alkyl chain (Figure 1d) or *p*-phenylene chains (Figure 1e)) connected to a hydrophilic dendron can be classified as dendritic amphiphiles [32,33,41,42,43,44,45,46,47].

This review will focus on amphiphilic dumbbells and amphiphilic JDs that self-assemble as vesicular structures. Readers that are interested in the self-assembly of the other members of the dendritic family are referred to other reviews and references [5,48,49,50,51,52,53,54,55,56].

### 2.1. Janus Dendrimers

**Figure 2:**
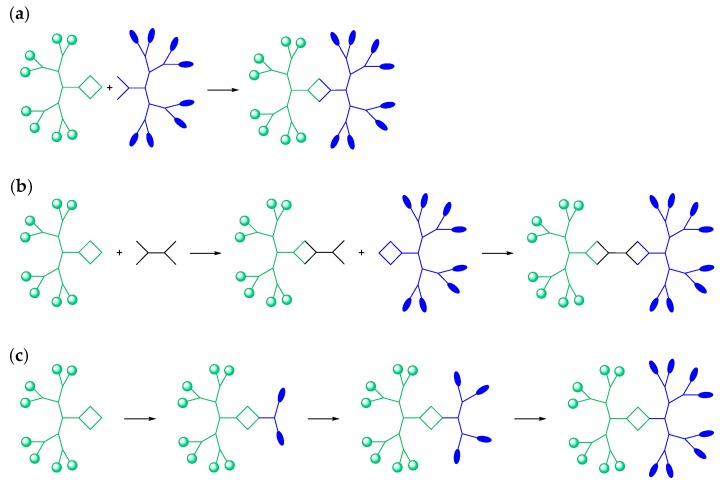
Main synthesis methods for amphiphilic JDs: (**a**) convergent method with two complementary dendrons; (**b**) convergent method with two dendrons complementary coupled to a multifunctionalized core; and (**c**) divergent method.

Janus dendrimers (JDs) are a structural class of dendritic macromolecules consisting of two dendrons, that either only differ in their end groups or completely in their structure. Their name is derived from the name of the two-faced Roman god of the beginnings and endings—Janus. By coupling dendrons of different polarities, one can take advantage of both the properties of dendrimers (i.e., defined structure and possibility of diversified functionalization) and the surface active and self-assembling features of amphiphiles in a single molecule [22]. In contrast to other amphiphilic polymeric structures, the structure of JDs can be precisely controlled: their size, architecture, density, generation and the number of end groups of the individual dendrons can be modified as desired by using standard organic chemistry. Amphiphilic JDs have been prepared by a variety of synthetic approaches [22]. The most used strategy is based on the convergent method and consists of coupling the different functionalized dendrons at their complementary cores (Figure 2a) [21,32,58]. Another possibility is the use of a multifunctionalized core (Figure 2b) [59,60]. After coupling with a dendron molecule, the free functional groups of the core are linked with other dendrons. A multifunctionalized core with an internal mirror plane even allows for simultaneous coupling of dendrons with the core [61,62]. The third alternative is the divergent method (Figure 2c). This rarely applied method uses the focal point of a dendron for the growth of new dendritic branches [63,64]. Details and other examples regarding these strategies can be found in other reviews and are not within the scope of this publication [22,23].

**Figure 3:**
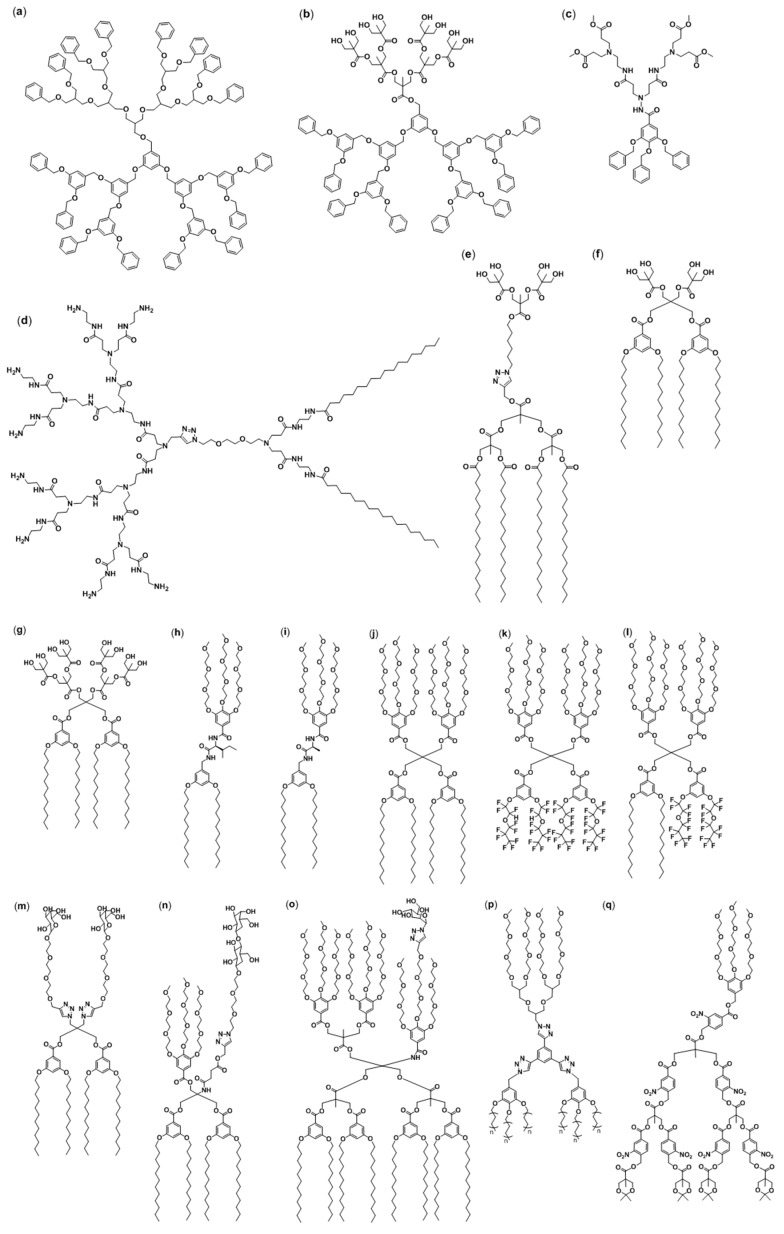
Structures of Janus dendrimers that form dendrimersomes: in organic solvents (**a**,**b**,**p**); and in water (**c**–**q**) (for (**p**), only by coassembly with nanoparticles).

Because of their amphiphilic nature, amphiphilic JDs are suitable building blocks for self-assembled structures such as vesicles. Their synthetic flexibility enables to easily adjust the hydrophilic-hydrophobic ratio to obtain the desired morphology. In 2005, 14 years after the first report about amphiphilic dendrimers by Newkome [29], the first example of JD self-assembly into vesicles was published [23]. Yang et al. reported the formation of vesicles in an organic solvent, from a JD based on a poly(benzyl)ether and a polyether dendron (Figure 3a). Few other examples of JDs forming vesicles in organic solvents can be found in the literature (Figure 3a,b,p, Table 1). However, since the application of vesicles in organic solvents is limited, most of the efforts in the field of DSs have been focused on the formation of vesicles from amphiphilic JDs in aqueous media.

An important step in this direction has been made by Percec et al. [21]. In a seminal paper, the authors reported the synthesis of 108 amphiphilic JDs and studied their self-assembling behavior in bulk and aqueous solution [21]. Thanks to this study and others, numerous hydrophilic (Table 2) and hydrophobic dendrons (Table 3) have been used for the construction of amphiphilic JDs forming vesicles in aqueous media. Hydrophilicity is typically introduced with peripheral oligoethylene oxide chains (Figure 3h [65], Figure 3i [66], Figure 3j,k,l [67], Figure 3p [68] and Figure 3q [63]), aliphatic polyethers (Figure 3a), polyamidoamines (PAMAM) (Figure 3c [69] and Figure 3d [32]), quaternary ammonium salts and hydroxy-terminated units such as dimethylolpropionic acid (bis-MPA) (Figure 3e [58], Figure 3f [70] and Figure 3g [71]), glycerol, or thioglycerol and saccharides such as d-mannose, d-galactose or d-lactose (Figure 3m [72], Figure 3n [73], Figure 3o [74]). Compared to the repertoire of hydrophilic dendrons, the hydrophobic part is less diversified. Typical hydrophobic moieties include simple aliphatic (Figure 3d [32], Figure 3e [58], Figure 3f [70], Figure 3g [71], Figure 3h [65], Figure 3i [66], Figure 3j [67], Figure 3m [72], Figure 3n [73], Figure 3o [74] and Figure 3p [68]) and fluorinated chains (Figure 3k,l [67]), poly(benzyl ethers) (Figure 3a [75], Figure 3b [76] and Figure 3c [69]) and poly(benzyl esters) (Figure 3q [63]).

Amphiphilic JDs can generally be divided into single–single and twin–twin JDs [21,66]. The labeling refers to the number of hydrophobic and hydrophilic dendrons used to synthesize the amphiphiles: while single–single molecules only consist of one hydrophobic and one hydrophilic building block, their twin–twin analogs are built up of two hydrophobic and two hydrophilic dendrons. Compared to the synthesis of twin–twin JDs, the synthesis of the single–single JDs involves less synthetic steps and is therefore less time-consuming. Pentaerythritol is usually used as core for twin–twin JDs and ester, amide, amine, azide, ethylene glycol, amino acids or perylene bisimide dye cores for single–single JDs (Table 4) [21,62,64,65,66]. Table 2, Table 3 and Table 4 present the various structural parts that can be found in JDs forming DSs in aqueous media.

To further extend the application range of DSs, various amphiphilic JDs were prepared comprising particular structural components, such as carbohydrates, photodegradable units, dyes or covalently conjugated contrast agents. A more detailed insight will be provided in the section about biomedical applications.

### 2.2. Amphiphilic Dumbbells

Another class of amphiphilic molecules that can be used for the formation of vesicles are amphiphilic dumbbells. These molecules consist of flexible dendritic branches covalently linked to both ends of a rigid elongated aromatic rod segment [34,35,37,38,39,40,50,82,83]. Their synthesis generally consists of coupling a dendritic wedge to one side of the rigid core and then connecting the other dendron with the remaining functional group of the core.

Amphiphilic dumbbells were first introduced in 2002 by Lee et al. They were a further development of the previously presented rod-coil block systems, where a dendritic wedge is grafted to only one side of the rod-building block, resulting in tree-shaped molecules [36,84,85]. The dendritic branches can both be hydrophilic, resulting in symmetric amphiphilic dumbbells. By replacing one hydrophilic wedge by a hydrophobic one, asymmetric amphiphilic dumbbells are formed. Common rod segments consist of hydrophobic *p*-phenylene or *p*-biphenylene chains [34,35]. Chiral or achiral aliphatic oligoether blocks are typically used as hydrophilic wedge and branched aliphatic chains as hydrophobic flexible wedge [34,35,40].

**Figure 4:**
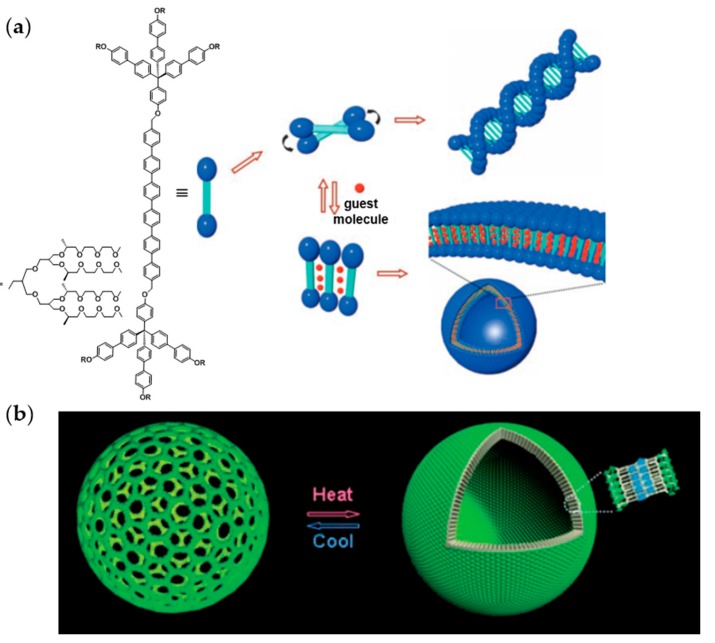
(**a**) Self-assembly of symmetric amphiphilic dumbbells in aqueous solution: helical nanofibers without guest molecules or vesicles after addition of guest molecules [34]; (**b**) Temperature-induced reversible closing of pores in a vesicle membrane [40]. Copyright Wiley-VCH, adapted from Angewandte Chemie International Edition 2006 [34] and reprinted from Angewandte Chemie International Edition 2008 [40].

The formation of supramolecular structures from amphiphilic dumbbells is generally induced by directly dissolving the amphiphile in an aqueous solution, which is a selective solvent for the hydrophilic (oligoethylene) dendrons. Symmetric amphiphilic dumbbells have been found to form helical nanofibers in aqueous solution (Figure 4a) [39]. In this specific case, the hydrophobic aromatic cores are surrounded by hydrophilic flexible segments that are exposed to the aqueous environment [34]. Interestingly, the addition of a hydrophobic guest molecule led to a reversible transformation of the helical structure into vesicles (Figure 4a) [34]. This morphological transition is caused by a packing rearrangement of the rod building blocks from twisted to parallel allowing more space for the hydrophobic guest molecules between the rods. The morphological transition into vesicles has been evidenced by encapsulation experiments with calcein and TEM measurements. The propensity of asymmetric dumbbell-shaped amphiphiles to form vesicular structures has been found to depend on the rod length, the length of the hydrophobic alkyl chains, as well as on temperature [34,35,37].

The use of stimuli-responsive vesicles as transport system is particularly interesting regarding the triggered release of cargo by external stimuli, such as temperature, pH, light, etc. Oligoether dendrons displaying a lower critical solution temperature (LCST) imparted thermoresponsive behavior to the amphiphilic dumbbells. An increase of the temperature above the LCST has been found to provoke a morphological transition from planar sheets to vesicles. This structural change can be ascribed to the dehydration of the oligoether dendrons resulting in an alteration of the hydrophilic-hydrophobic ratio [35,40]. A temperature-induced reversible closing of pores inside a vesicle membrane has been observed by Kim et al. (Figure 4b) [40]. Upon heating above the LCST, the pores closed with preservation of the vesicular structure. The triggered release of cargo was confirmed by fluorescence experiments with calcein and intracellular delivery experiments of a fluorescently labeled DNA oligomer [40].

Despite all these fascinating features, vesicles formed by asymmetric amphiphilic dumbbells generally exhibit sizes between 100 nm and 1 µm and a rather broad size distribution. However, vesicles having diameters below 100 nm are preferred for biomedical applications, e.g., in the field of drug delivery, since smaller vesicles display a longer blood circulation than larger vesicles (d > 200 nm) [86,87,88,89,90,91,92]. Furthermore, a narrow size distribution is an important factor as it has an impact on the pharmacokinetics of the vesicles [92].

## 3. Vesicles Formation

### 3.1. Non-Covalent Interactions and Geometrical Considerations

The self-assembly process is governed by various non-covalent interactions between amphiphiles and their environment. Hydrogen bond formation and the hydrophobic effect are the main forces that drive amphiphile self-assembly [34]. The stabilization of the structures is associated with an enthalpic and entropic gain [34]. Other interactions in the self-assembly process are π–π interactions, van der Waals interactions and electrostatic interactions. Amphiphilic JDs do not only self-assemble into vesicular structures. Cryo-TEM measurements showed that they can also form other aggregates, such as cubosomes, spherical or cylindrical micelles, solid lamellae, organogels, hydrogels, etc. [21,69,72,93]. The shape and size of the resulting aggregates depend on preparation conditions (concentration, temperature, solvent, pH and ionic strength) and, mainly, on the geometry of the amphiphile. The latter can vary among conical, cylindrical and inverted conical depending on the ratio between the hydrophilic and hydrophobic part. According to these geometrical considerations, different aggregates can result [2,94]. The influence of the molecular geometry on the aggregate structure can be described by the critical packing parameter introduced by Israelachvili et al. for surfactants [94]. This parameter comprises the hydrophobic group volume, the hydrophobic group length as well as the equilibrium surface area per molecule at the aggregate interface (related to the hydrophilic head group).

**Figure 5:**
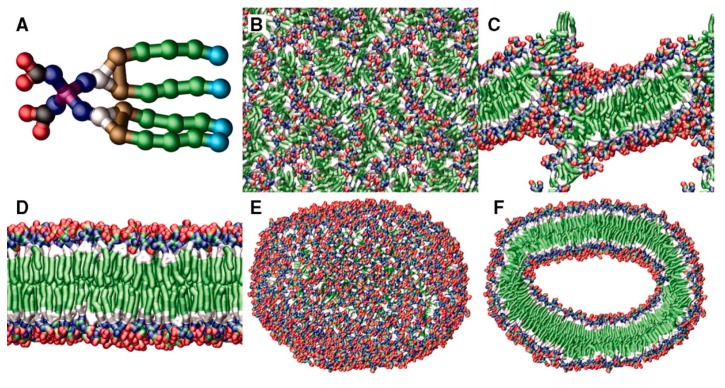
Self-assembly of the Janus dendrimer (3,5)12G1-PE-BMPA-(OH)_4_ using CG-MD simulations. (**A**) Molecular model used for the simulations; (**B**–**E**) CG-MD simulations starting from a fully random initial configuration of JD molecules in aqueous solution. Vesicle formation is completed after a simulation time of 80 ns; (**F**) Cut away view of (**E**) showing the hollow core of the vesicle. Copyright American Association for the Advancement of Science, reprinted from Science 2010 [21].

However, predicting the morphology of self-assembled structures based on a net geometrical consideration is a simplified model. Local interactions between the amphiphile and its surrounding (i.e., solvent molecules) are not considered even though they play an important role in the self-assembling process [69,95]. With the help of coarse-grained molecular dynamics (CG-MD) simulations, such interactions are included, enabling a detailed insight into the process. CG-MD simulations have been performed by Percec et al. for the aggregation of a JD consisting of four terminal C12 chains for the hydrophobic part and four bis-MPA functionalities as end groups of the hydrophilic part [21]. Depending on the quantity of CG JDs molecules (from 216 to 13.824 CG JDs molecules) and on the hydration level (from 41 CG water molecules per CG JD molecule to 269 CG water molecules per CG JD molecule), the formation of vesicles, bicontinuous phases or disk-like micelles was predicted. Figure 5 shows a series of snapshots from a CG-MD simulation that starts with a random configuration of monomers and ends with the formation of a unilamellar vesicle within a few (80 ns) nanoseconds.

### 3.2. Classification of Dendrimersomes

**Figure 6:**
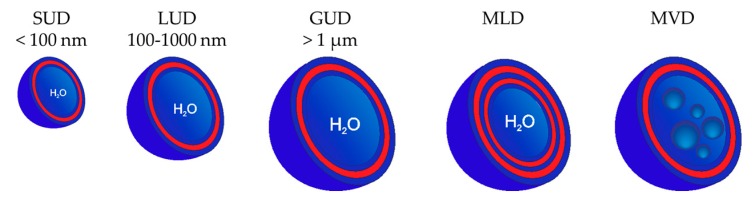
Classification of vesicles according to their size and number of bilayers.

In general, vesicles are classified according to their size or the number of bilayers they consist of (Figure 6) [1,96]. Unilamellar dendrimersomes are composed of only one bilayer, while multilamellar dendrimersomes (MLD), also called “onion-like” dendrimersomes, have several concentric bilayers. In some cases, smaller vesicles can be contained in one large dendrimersome. Those vesicles are called multivesicular dendrimersomes (MVD).

Based on their size, unilamellar dendrimersomes are classified as small unilamellar dendrimersomes (SUD), large unilamellar dendrimersomes (LUD) and giant unilamellar dendrimersomes (GUD). SUDs have diameters less than 100 nm, while LUD sizes are between 100 nm and 1000 nm. Vesicles bigger than 1 µm are classified as GUDs [97].

### 3.3. Preparation Methods for Dendrimersomes

Many preparation methods for liposomes have been reported in the literature [1,98], some of which are used for DSs preparation. Like their lipid counterparts, amphiphilic JDs must be in contact with an aqueous phase to form supramolecular nanostructures, such as DSs. The particle formation can be done in the presence or absence of an organic solvent. In the latter case, the amphiphiles are directly exposed to the aqueous medium in a dry state. If an organic solvent is used to facilitate the self-assembly, the amphiphile is first dissolved in the solvent and then mixed with the aqueous solution. The solvent is eventually removed afterwards.

**Figure 7:**
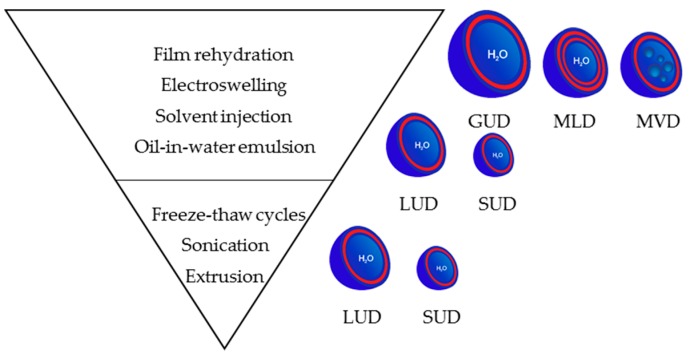
Overview of the different methods and post-preparation treatments for generating dendrimersomes of different sizes and lamellarity.

In the following section, the most important preparation methods for DSs are described. Figure 7 gives an overview of the preparation methods and the resulting DSs.

#### 3.3.1. At the Microscale: Giant Vesicles

##### Preparation by Film Rehydration Method

**Figure 8:**
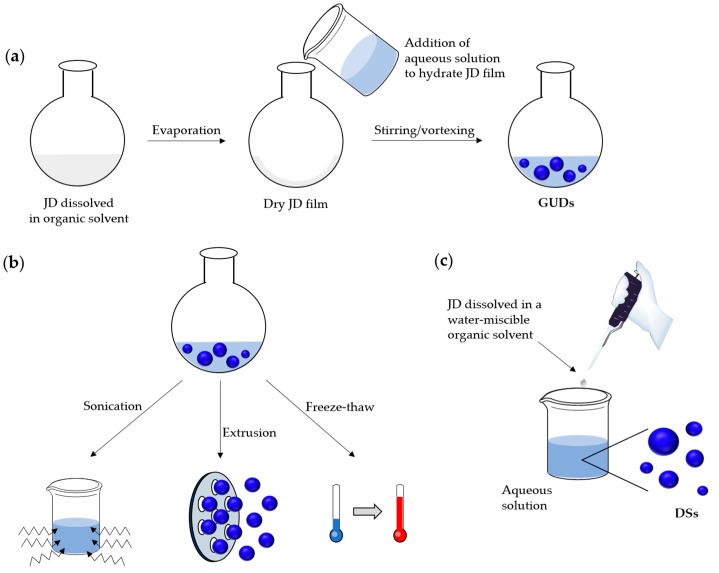
(**a**) Film rehydration method for the preparation of GUDs; (**b**) post-preparation methods to control the size, size distribution and lamellarity of dendrimersomes: sonication, extrusion, and freeze–thaw cycles; and (**c**) preparation of DSs by solvent injection method.

The preparation of GUD by means of the film rehydration method has been adapted from the preparation of giant liposomes [99,100]. In this method, the JD is dissolved in a volatile organic solvent and spread on a solid surface (Figure 8a). After evaporation of the solvent, the amphiphilic JDs are organized in a multi-stacked bilayer film on the surface. By hydrating this film, water molecules penetrate between the stacks and the bilayers start to separate out. Vesicle formation occurs above the so-called solid-ordered (s_o_)/liquid-disordered (l_d_) or gel/liquid crystalline main phase transition temperature [101]. Hence, the hydration step is typically carried out at elevated temperatures (50–60 °C) so that the bilayers are in a liquid-disordered state. The importance of the temperature has already been proven for liposomes [102]. A study from Hishida et al. showed that the capacity for giant vesicles formation depends on the phase of the lipids: only hydration of smooth, flat bilayers in the l_d_ phase resulted in giant vesicles, whereas layers in the s_o_ phase did not form them [102].

Encapsulation of hydrophilic or hydrophobic cargo (e.g., dyes, drugs, contrast agents) can be performed during the formation of GUD by this method [21,64]. The hydrophobic component is dissolved in the organic phase containing the amphiphile, while the hydrophilic cargo is dissolved in the aqueous solution used for the hydration step. Efficient coassembly of different types of JDs (fluorinated and hydrogenated) has also been achieved recently with this method [67].

##### Preparation by Electroswelling Method

The electroswelling method is another method to generate GUDs. It relies on the hydration of a dried JD film in the presence of an externally applied electric field. Although this method is commonly used for liposome formation, it is rather rarely encountered in DSs preparation [103,104,105,106]. The GUDs are formed in an aqueous solution, when a low-frequency alternating electric (AC) field is applied onto a conductive surface (indium tin oxide (ITO) coated glass) with a JDs film. The mechanism of this method is at present not fully understood. It is proposed, however, that the electric field causes an electroosmotic flow of the aqueous phase, which results in mechanical shearing of the swollen JDs film [107]. By this means, vesicle formation is induced through bilayer destabilization and fusion of adjacent vesicles [104,105,106].

#### 3.3.2. At the Nanoscale

##### Preparation by Film Rehydration Method Followed by Post-Preparation Steps

The GUDs prepared by the film rehydration method can be subjected to post-processing steps to obtain LUDs and SUDs. In addition to the size-control, polydispersity as well as lamellarity of the vesicles can be influenced.

LUDs can be obtained by sonication of GUDs suspensions (Figure 8b). By using acoustic energy, sonication induces pressure stress that breaks up the large vesicles into smaller ones. The duration of the treatment and the power input have an influence on the size of the processed vesicles. Higher input results in greater shear forces within the solution, resulting in a decrease of the vesicle size and polydispersity [108].

The extrusion method is commonly applied for the preparation of SUDs (Figure 8b). In this method, a dispersion of vesicles is pressed several times through a membrane with small pores. The process should be carried out above the phase transition temperature to prevent bursting of the filters. Until today, the exact mechanism of extrusion has not been elucidated. Several studies based on the extrusion of MLVs made from lipids, such as POPC (1-palmitoyl-2-oleoyl-*sn*-glycero-3-phosphatidylcholine) or egg PC (egg phosphatidylcholin), have been conducted to provide new insights into the process [109,110,111,112,113]. For example, Thompson et al. suggested a break-up mechanism based on the Rayleigh instability, which predicts that cylindrical soap films rupture into droplets because of surface tension [114]. Bruinsma postulated that MLVs deform inside pores into long cylindrical structures of radius R. They then rupture either inside the pore or at the pore exit into smaller cylinders of length λ, where λ = 2πR, and finally reform into vesicles [109]. Patty et al. proposed a model based on blowing bubbles, where MLV block the entrance to the pores. As the applied pressure induces a surface tension in the bilayer that exceeds the lysis tension of the membrane, vesicles break-up [112].

Studies on parameters affecting the size of extruded MLVs revealed pressure-dependency of the vesicle size: with increasing pressure, the final mean size of the produced vesicles decreases [111,112]. For example, the average radius of POPC vesicles extruded through a membrane with a nominal pore diameter of 100 nm, decreases from 70 nm at 50 psi to 59 nm at 400 psi. Additionally, the resulting vesicle size has been found to depend on the pore size of the membrane: larger pores generally yield vesicles only slightly larger than the pore size, while smaller pores lead to vesicles larger than the pore size [110]. Further investigations showed that multiple extrusion cycles are required to reduce the size and multilamellarity of MLVs [113]. In contrast thereto, a study from Jadhav et al. showed that SUVs with low polydispersity can also be generated by a single-pass extrusion of GUVs at extrusion pressures lower than those required for the preparation of SUVs from MLVs [115]. Their size depended primarily on the suspension velocity within the membrane pore.

In the case of MLD formation, freeze–thawing cycles can be applied prior to extrusion to reduce the polydispersity and lamellarity (Figure 8b). Moreover, a freeze–thaw pretreatment can be used to promote drug-encapsulation as the disruption of the bilayer allows drug molecules to diffuse into vesicles. A freeze–thaw cycle consists in freezing the vesicle suspension and subsequent thawing at a temperature above the phase transition temperature of the JDs. Because of ice crystal formation during the freezing process, bilayers are damaged and reassemble afterwards to new vesicles. Freeze–thawing was demonstrated to cause fusion of single unilamellar vesicles necessitating further homogenization steps such as extrusion [116,117].

##### Solvent Injection Method

A simple method for the preparation of DSs (and glycodendrimersomes—GDSs—dendrimersomes formed from Janus dendrimers containing carbohydrate moietie(s) also called Janus glycodendrimersomes (JGDs)) is the solvent injection method sometimes also referred to as nanoprecipitation method (Figure 8c). It has been applied for the preparation of SUDs, onion-like DSs or even giant DSs [72]. In this method, JDs are dissolved at a desired concentration in a water-miscible organic solvent that is a good solvent for the hydrophilic and hydrophobic part. Depending on the JD structure, polar protic (ethanol, isopropanol, etc.) or polar aprotic (THF, acetone, acetonitrile, 1,4-dioxane, DMSO, etc.) solvents can be chosen as good solvents [21]. While either EtOH or THF were chosen as good solvent for JDs, THF was preferred for JGDs. A given volume of the organic phase containing the amphiphile was then injected into an aqueous solution and mixed rapidly. The use of buffers, such as phosphate-buffered saline (PBS) or 4-(2-hydroxyethyl)-1-piperazineethanesulfonic acid (HEPES), instead of water enables to simulate physiological conditions and to avoid changes in pH. As the solvent quality changes from unselective to selective, the self-assembly process is initiated: the amphiphilic molecules precipitate and form planar bilayer fragments [118,119,120]. These fragments form vesicles to minimize the contact of the hydrophobic parts with the aqueous phase. The size of the resulting DSs depends on the starting JDs concentration in the organic phase, the end concentration of the JDs in the aqueous phase or the nature of the common and selective solvent [21]. No investigations concerning the impact of the injection velocity on the vesicle size have been conducted so far.

Even though the solvent injection method is simple and less time-consuming compared to other preparation methods, the use of an organic solvent is a major drawback for future biomedical applications: the removal of solvent is indispensable, as residual solvent in the final DSs solution may be toxic and thus hazardous to human health. Moreover, remaining solvent may affect the chemical structure of the entrapped substance and influence the stability of the vesicles [121,122]. Although several removal techniques, such as dialysis or gel filtration, are available, traces of solvent often remain in the final formulation. The choice of ethanol as common solvent represents a compromise, as it is biocompatible and largely used in pharmaceutical preparations [123,124].

The self-assembly can also be initiated by addition of the selective solvent to the good solvent phase containing the amphiphilic JDs (reverse injection). The opposite order of addition generally leads to larger DSs [63,65]. A similar outcome was observed for polymersomes [125,126]. Compared to the direct injection, the reverse injection enables JDs to diffuse over a longer period of time to form vesicles. In this case, the diffusion velocity and thus DSs size is influenced by the nature of common solvent, the miscibility between the phases and the viscosity [63]. In contrast to the size, the order of addition does not affect the lamellarity of the vesicles, as both direct and reverse injection were able to generate onion-like DSs [65].

##### Emulsion

The oil-in-water (o/w) emulsion solvent evaporation technique is a less common alternative to the previously presented methods for the formation of LUDs [58,63]. In this method, the amphiphile is first dissolved in a water-immiscible solvent (e.g., dichloromethane) and then emulsified in water under stirring. Solvent evaporation results in an aqueous suspension containing LUDs. Encapsulation of a hydrophobic drug can also be achieved by dissolving the drug in the organic solvent [58].

Despite the various types of preparation methods, a common problem which arises is the difficulty of upscaling these methods. If the targeted applications of DSs are in the biomedical field, their production in large quantities must be feasible. In addition, the economical aspect as well as reproducibility from batch to batch must be considered. A possible answer to these requirements could be the use of microfluidic approaches, which have already shown promising results for liposomes and polymersomes [127,128,129,130].

### 3.4. Parameters Influencing the Formation, Structure, Stability and Biocompatibility of Vesicles

#### 3.4.1. Formation of Vesicles

##### Influence of JD Structure

Molecular parameters, such as the hydrophilic-hydrophobic ratio of the amphiphile, should be considered for the generation of DSs. Depending on this ratio, the interface between the hydrophobic and hydrophilic part varies and thus the resulting curvature of the self-assembled structure. The hydrophilic-hydrophobic ratio can be varied by using dendrons of various generations or even by coassembling amphiphiles of different shapes [61,62,63]. Furthermore, the ability to form DSs can be linked to structural characteristics. For instance, studies showed that the aggregation behavior of single–single JDs is influenced by the substitution pattern of their hydrophilic part: only JDs with a (3,4,5)-substitution with tri(ethylene glycol) monomethyl ester chains favored the formation of DSs [66]. For JGDs the formation of (soft) DSs was linked to given structural features such as a tri- or tetra-ethylene glycol spacer attached to the carbohydrate and a (3,5)-substituted hydrophobic dendron with linear or branched alkyl chains [72].

##### Solvent Influence

**Figure 9:**
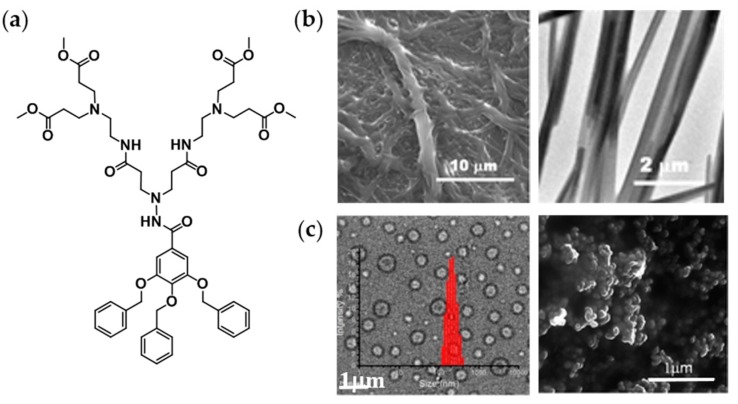
(**a**) JD from poly(aryl ether) linked PAMAM; (**b**) SEM (**left**) and TEM (**right**) images of xerogel of the amphiphile in DMSO/water mixture; and (**c**) TEM images superimposed with DLS data (**left**) and SEM images (**right**) of vesicles in EtOH/water mixture. Copyright Wiley-VCH, reprinted from ChemistrySelect 2016 [69].

The choice of the common solvent, dissolving both the hydrophilic and hydrophobic part of the amphiphile, can influence the morphology and size of the resulting aggregates. Prabakaran et al. demonstrated the possibility to control the self-assembling process of a PAMAM-based amphiphilic JD (Figure 9a) using solvents with different hydrogen bonding donor ability [69]. While vesicles were generated in an ethanol/water mixture (Figure 9c), long fibers (gels) were obtained in a DMSO/water mixture (Figure 9b). Vesicle formation was ascribed to the protic nature of ethanol: solute–solute and solute–solvent hydrogen bonds compete and thus prevents the formation of long-ranged self-assembled structures such as fibers. A similar trend was observed for the formation of onion-like DSs [65]. Amphiphilic JDs containing an amide-core favoring hydrogen bond formation (Figure 3h) assembled as multilayered vesicles. Vesicles of spherical shape with uniform spacing between the bilayers were only attainable with non-protic solvents, whereas the use of alcohols yielded irregular vesicular structures with random distance between the layers [65]. In this case, the competition between solute–solute and solute–solvent hydrogen bonds seems to play a significant role on the self-assembly process as well. The influence of the organic solvents nature on polymersomes size has been found to be significant when the organic solvent is added in water [125,126]. Such an impact was not observed for DSs formation. Although DSs of different sizes could be obtained using protic and aprotic solvents, there was no evident correlation between the polarity of the solvent or the hydrogen bonding donor ability and the resulting size [21,63]. However, it was obvious that the vesicle size can be tuned by the rate of formation using organic solvents of different nature. In general, the use of moderately polar solvents, such as THF or CH_2_Cl_2_, generally led to vesicles with bigger sizes and polydispersities. Dissolving the amphiphiles directly in a nonpolar solvent can induce the formation of inverted vesicles. In this case, the impact of the shape of the JD on the resulting structure is prevalent as well [68]. The choice of an appropriate common solvent is indispensable: if the amphiphile is not molecularly dissolved, a crystalline membrane can result and thus the formation of hard dendrimersomes [72].

The choice of the aqueous selective solvent can also be used as tool to vary the size of the DSs [72,78]. For instance, the dimensions of GDSs assembled from the same JGDs were shown to be generally smaller in water and PBS than those in HEPES. The same trend was observed for onion-like DSs assembled from single–single JDs [74].

##### Critical Aggregation Concentration

The critical aggregation concentration (CAC) is an important parameter that provides information about the stability of amphiphilic aggregates. Amphiphiles with low CACs have slow chain exchange dynamics. This means that the probability that the amphiphiles go into solution and interchange with other structures formed is very low. Consequently, their assemblies have slow rates of dissociation, which is very important in view of application as drug delivery systems. Compared to lipids, amphiphilic block copolymers have significantly lower CACs [131,132,133]. 

Thus far, investigations concerning the CAC of amphiphilic JDs and the factors influencing it were not put to the foreground. Concentrations for DSs and GDSs preparation can vary between 0.025 mg/mL and 10 mg/mL, while typical concentrations range between 0.5 mg/mL and 1 mg/mL [21,66,72,78].

#### 3.4.2. Size of Vesicles and Membrane Characteristics

##### Membrane Thickness: Influence of JD Structure and Interdigitation of Its Hydrophobic Part

**Figure 10:**
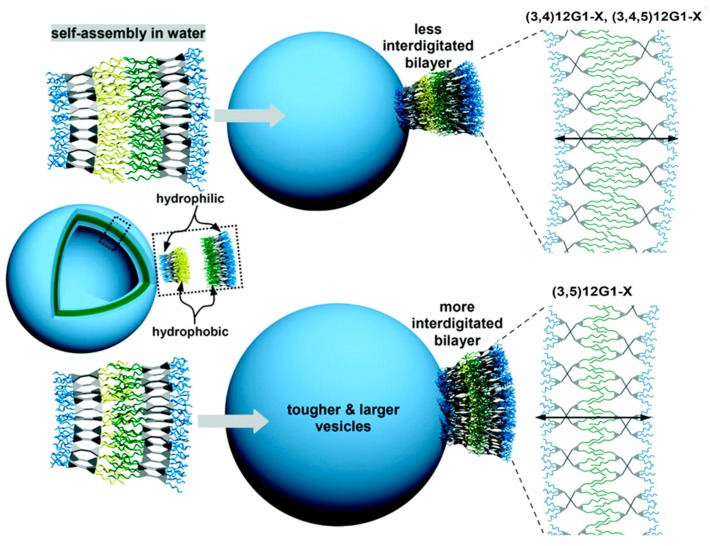
Correlation between substitution pattern of the hydrophobic part of hydrogenated JDs, its interdigitation in the bilayers and the vesicular membrane thickness, curvature and size. Copyright American Chemical Society, adapted from Journal of the American Chemical Society 2011 [77].

DS membranes are thinner compared to polymersomes, with dimensions similar to that of biological membranes (4–7 nm) [21,66,67,77]. The key factor influencing the membrane thickness is the molecular structure of the JD, more precisely of its hydrophobic part [77]. A correlation between the hydrophobic substitution pattern of hydrogenated JDs and the d-spacing value in the bulk-phase (membrane thickness) obtained by X-ray diffraction (XRD) has been found: JDs with (3,4)- and (3,4,5)-hydrophobic pattern possess thicker membranes (up to 7 nm), than those with (3,5)-substitution (around 5 nm) [77]. The smaller membrane thickness was attributed to the stronger interdigitation of the aliphatic chains, which are more separated in (3,5)-JDs than in (3,4)-JDs (Figure 10). Fluorinated JDs (Figure 3k) showed a slightly different trend [67]. Chains were interdigitated in DSs formed from JDs with (3,4)- and (3,5)-substitution pattern. This was not the case for DSs from JDs with (3,4,5)-substitution pattern. Furthermore, direct comparison of fluorinated and hydrogenated interdigitated membranes revealed that fluorinated membranes were thinner than the hydrogenated ones (4.7 nm and 5.0 nm for fluorinated membranes compared to 6.1 nm for hydrogenated membranes). This was explained by the shorter length of the fluorocarbon chains compared to the alkyl chains. On the other hand, neither the substitution pattern nor the generation of the hydrophilic dendron seemed to have a specific influence on the membrane thickness [77].

##### Curvature/Size: Influence of Hydrophilic Part, Core of the JDs and Concentration of the Solution

As already described in the previous paragraph, the hydrophobic substitution pattern of single–single and twin–twin JDs influences the membrane thickness [77]. As the membrane thickness affects the vesicular curvature, it has an impact on the DSs size: thin membranes generated by the interdigitation of the hydrophobic chains resulted in a lower DSs curvature, ergo in bigger vesicles (Figure 10).

When the hydrophilic part consists of charged end groups at neutral pH (e.g., amines or carboxylic acids), this presents a further influencing parameter on the size of the corresponding DSs. Their electrostatic interactions can vary as a function of the nature or ionic strength of the selective solvent as seen for polyion complex vesicles (PICsomes) [134,135]. Hence, the dimension of the hydrophilic part can change and consequently the entire size of the vesicle. Interaction of the JD with siRNA can even induce a morphology change [32].

Another structural component that has an impact on the vesicle size is the core of JDs [66]: JDs with cores that can form hydrogen bonds (e.g., amide core) assemble into smaller vesicles compared to those with cores that cannot create hydrogen bonds (e.g., ester core). The authors suggested that hydrogen bonding prevents the interdigitation of the aliphatic chains in the membrane and thus results in smaller DSs.

**Figure 11:**
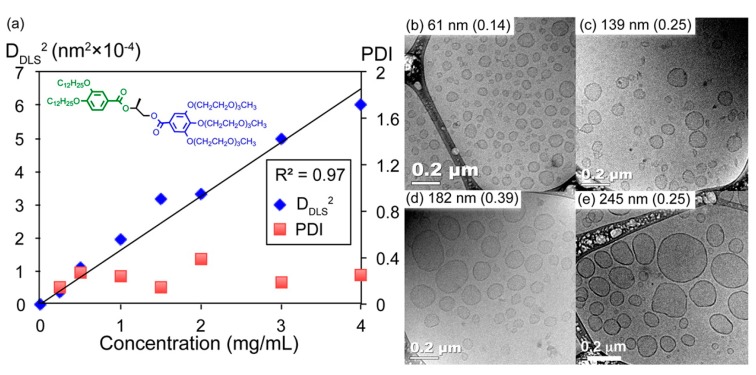
Variation of the diameter of dendrimersomes as a function of the final JDs concentration. (**a**) Square diameter (*D*_DLS_^2^)–concentration relationship of the dendrimersomes formed by (3,4)12G1-I-PPD-(3,4,5)-3EO-G1-(OCH_3_)_3_. Representative cryo-TEM images of the dendrimersomes with the indicated diameter (*D*_DLS_^2^, in nm) and polydispersity index (PDI, in parentheses) at a final concentration of: (**b**) 0.25 mg/mL; (**c**) 1 mg/mL; (**d**) 2 mg/mL; and (**e**) 4 mg/mL. Copyright American Chemical Society, reprinted from ACS Nano 2014 [66].

The size of DSs (unilamellar as well as onion-like) can be controlled by changing the final concentration of the JDs in the aqueous solution: an increase of the concentration increases the vesicle size (Figure 11) [21,65,66,67,72,73,74,77]. However, the range in which variation of the size is possible is controlled by the curvature limit. The concentration-size correlation, already proven for polymersomes and liposomes, can be explained by the fact that the concentration of JDs controls the number of monomers forming a single vesicle [134,136,137]. A linear correlation between the DSs/GDSs size (square of diameter) and the concentration was found for single–single, twin–twin DSs as well as JGDs [66,74,77]. For GDSs, the nature of the carbohydrate as well as the incorporation of a spacer can have an influence on the size [72,73,74]: GDSs containing d-lactose were in general smaller than those with d-mannose. A spacer generally decreased the vesicle size, however no specific correlation between the spacer length and the size seemed to exist.

Incorporation of different membrane components can affect the DS size in different ways [70,74]: the reconstitution of the membrane protein complex called reaction center (RC) for example resulted in smaller vesicles, compared to the one without guest protein (990 nm without membrane protein compared to 300 nm with membrane protein) [70]. On the other hand, coassembly of JDs with components of bacterial membrane vesicle (BMV) did not change the size compared to that of the simple DSs [74].

##### Membrane Fluidity: Influence of Hydrophobic Part

The membrane fluidity of DSs can be influenced by the nature of the hydrophobic part. For example, incorporation of fluorinated alkyl chains promotes the formation of soft DSs, while their hydrogenated counterparts only form hexagonal phases [67]. This can be explained by the high fluidity of fluorocarbons.

Another influencing factor is the substitution pattern of the hydrophobic segment [21,77]. Differential scanning calorimetry measurements revealed that JDs and JGDs with (3,5)- and (3,4,5)-substituted hydrophobic dendrons exhibit a phase transition at lower temperatures in bulk, resulting in the formation of soft DSs. A (3,4)-pattern is associated with higher phase transition temperatures and produces hard DSs. The core of a JD on the other hand does not have an impact on membrane fluidity. Comparison of JDs consisting of the same dendron scaffold but different cores only revealed an influence on the transition temperature but not on their ability to form soft DSs [66]. The impact of the hydrophobic branching pattern on the stability and mechanical properties of vesicles was demonstrated by micromanipulation experiments [21,77]. DSs formed by (3,4)- and (3,5)-dendrons were harder to break (elastic area expansion modulus K_a_ between 267.5 and 976 mN/m) or bend (lysis tension τ_s_ between 15.11 and 20.4 mN/m) than their (3,4,5)-homologs (K_a_ between 42.44 and 267.5 mN/m and τ_s_ between 0.88 and 12.7 mN/m). This was attributed to the stronger interdigitated arrangement of the alkyl chains. Comparative studies with liposomes and polymersomes proved the competitiveness of DSs. In general, their strength excels the one of polymersomes (K_a_ = 100 to 140 mN/m depending on the amphiphilic block copolymer) and unstabilized liposomes (K_a_ = 57 to 234 mN/m depending on the lipid).

##### Lamellarity: Influence of H-Bonding Moieties

**Figure 12:**
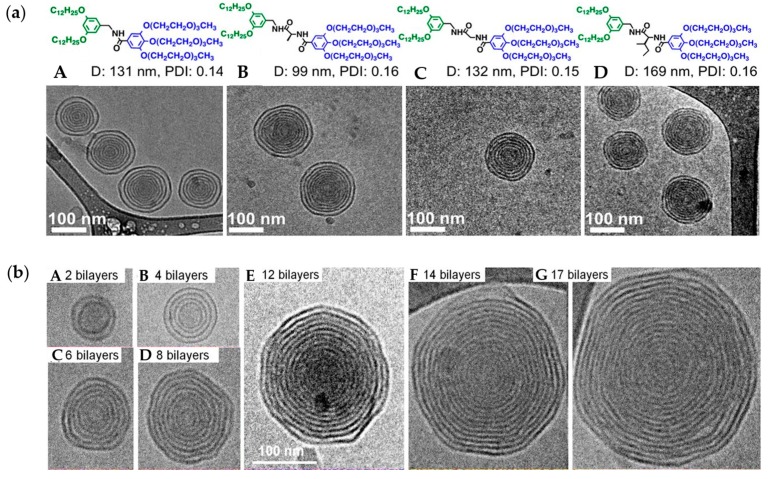
(**a**) Representative cryo-TEM images of onion-like vesicles self-assembled by injection of THF solution of: (**A**) (3,5)12G1-CH_2_-NH-(3,4,5)-3EO-G1-(OCH_3_)_3_; (**B**) (3,5)12G1-CH_2_-**l**-Ala-(3,4,5)-3EO-G1-(OCH_3_)_3_; (**C**) (3,5)12G1-CH_2_-Gly-(3,4,5)-3EO-G1-(OCH_3_)_3_; and (**D**) (3,5)12G1-CH_2_-**l**-Ile-(3,4,5)-3EO-G1-(OCH_3_)_3_ in water (1 mg/mL). (**b**) Cryo-TEM images of onion-like dendrimersomes self-assembled from (3,5)12G1-CH_2_-**l**-Ala-(3,4,5)-3EO-G1-(OCH_3_)_3_ in water at concentrations of: (**A**) 0.025 mg/mL; (**B**) 0.1 mg/mL; (**C**) 0.2 mg/mL; (**D**) 0.5 mg/mL; (**E**) 1 mg/mL; (**F**) 2 mg/mL; and (**G**) 2.5 mg/mL. Adapted from [65].

Amphiphilic JDs can self-assemble into unilamellar as well as onion-like DSs with identical spacing between the bilayers [21,65,66,67,77]. It turned out that onion-like structures were preferentially formed when the core of JDs consisted of a hydrogen-binding functionality (amide) (Figure 12a) [65]. JDs with ester cores either led to the formation of unilamellar or multivesicular vesicles. However, the presence of a H-bonding group is not necessary for the formation of multilamellar vesicles as evidenced by the formation of onion-like vesicles self-assembled from twin–twin fluorinated and fluorinated hydrogenated JDs [67]. This reveals the possible existence of other factors influencing the lamellarity of DSs. The number of bilayers (as well as the size) in onion-like DSs depends on the final concentration of the JD in solution: higher concentrations result in an increased number of bilayers (Figure 12b).

The formation of onion-like GDSs was found to be governed by the chemical nature of the carbohydrate in the hydrophilic part [74]. Only twin–mixed amphiphilic JGDs containing d-mannose self-assembled into onion-like vesicles [74]. Replacement of the d-mannose headgroup by d-lactose resulted in unilamellar vesicles, while d-galactose JGDs formed a mixture of unilamellar and onion-like vesicles. Dissipative particle dynamics (DPD) simulations of the self-assembly of amphiphilic Janus oligomers were performed to gain insight into the conditions favoring the formation of onion-like vesicles [138]. Hydrophilicity of the hydrophilic group has been shown to be one key parameter that can influence their formation. However, because of the complex structure of JDs, these simulations do not perfectly describe their self-assembly as they were based on a minimalist model, hence other factors may play a role.

#### 3.4.3. Biocompatibility and Stability of Vesicles

##### Biocompatibility

Only few studies about the biocompatibility of DSs can be found in the literature. Percec et al. evaluated the cytotoxicity of three DSs to human umbilical vein endothelial cells (HUVEC) and compared it to that of polyethyleneoxide-containing polymersomes [21]. The DSs used in the study were composed of twin–twin JDs which consisted of hydrophobic (3,4)-, (3,5)- or (3,4,5)-substituted benzylether dendrons with C12 chains and (3,4,5)-substituted benzylether dendrons with triethylene glycol chains. DSs showed similar cytotoxicity to HUVEC cells than polymersomes. Depending on the substitution pattern of the hydrophobic part, the cell viability after 4 h of incubation ranged between 40% and 60%.

DSs composed of a twin–twin JD bearing (3,5)-substituted benzylether dendrons with C12 chains and G2-bis-MPA dendrons and a small amount of the lipid DSPE-PEG-2000-COOH have been recently used for imaging applications [64,81]. In this context, cytotoxicity tests have been performed. Neither Gd-containing vesicles nor the unloaded ones affected the cell viability of fibroblasts or two populations of murine macrophages [64]. Even after 48 h of incubation and a high concentration of DSs (10 mg/mL) no negative effect was observed. Further tests with the dual-loaded DSs (Gd-complex and antitumor drug) demonstrated only a slight cytotoxicity for fibroblast, but a desired toxicity against HUVEC (anti-angiogenic effect) and melanoma cells (anti-tumor effect) after 24 h of incubation [81].

##### Stability Over Time and as a Function of Temperature, pH and Presence of Electrolyte

Physical stability of vesicles is an important factor when it comes to their use as drug delivery systems (DDS). The stability of DSs generally depends on the JD structure, physical variables, such as the temperature and on the composition of the solvent phase [21].

DSs prepared via ethanol injection method in water and stored at 25 °C were shown to have a good stability over time [21]. In some cases, the vesicles were stable up to 244 days (twin–twin JDs bearing G1 or G2 bis-MPA dendrons). GDSs also proved to be stable over time. In some cases, GDSs did not undergo a change in size over a testing time of 140 days [72,78]. Some of the GDSs were even stable in buffers, such as HEPES or PBS.

Investigations about the effect of temperature variations on DSs size and polydispersity index (PDI) revealed a temperature-dependent stability [21]. A study with two selected examples of twin–twin JDs only differing in their hydrophilic part (triethylene glycol chains vs. G2 bis-MPA dendron) showed that an increase of the temperature from 25 to 80 °C can have different effects on DSs. The size of DSs formed from twin–twin JDs with triethylene glycol chains increased with increasing temperature while maintaining a PDI of ~0.15, while DSs formed from twin–twin JDs with G2 bis-MPA dendron maintained a constant size of ~135 nm over the all temperature range but the PDI decreased (from ca. 0.08 to under 0.02).

Complex solutions, such as plasma, can affect the stability of vesicles due to the presence of proteins. In vitro stability tests of Gd-containing DSs (containing a small amount of the lipid DSPE-PEG-2000-COOH) in plasma-mimicking fluids were performed at 37 °C for 1 h [64]. DSs based on JDs with the (3,5)-pattern displayed a greater ability to retain their cargo than DSs made of the (3,4) analogs. Moreover, interestingly, the stability of DSs made of lower generation JDs (G0G1) was greater than the stability of DSs made of higher generation dendrons. Additional in vivo tests in mice revealed good stability of the most stable DSs of the in vitro studies (comparable to stealth liposomes) [64]. 

Finally, the stability of vesicles can be enhanced by incorporating polymerizable groups (double bonds) in the hydrophobic part of the amphiphile, enabling a covalent fixation of the bilayer structure via polymerization [62].

## 4. Biomedical Applications of Vesicles from Amphiphilic Dumbbells and Janus Dendrimers

Nanosized vesicles can be used as nanocarriers for drugs (therapeutic), imaging agents (diagnostic) or for a combination of both (nanotheranostics). They can also function as fluorescent pH sensors to discriminate for instance pH in vitro/in vivo or can be used as tools for understanding the role of membrane proteins or lectin recognition on cells.

At the microscale, dendrimersomes show great potential as artificial cells being able to integrate membrane proteins. These structures are also capable of self-sorting, which is found in Nature, for example in the case of proteins and DNA that seek out specific partners in high-fidelity recognition processes.

### 4.1. At the Nanoscale

#### 4.1.1. Vesicles from Amphiphilic Dumbbells as Fluorescent pH Sensor

**Figure 13:**
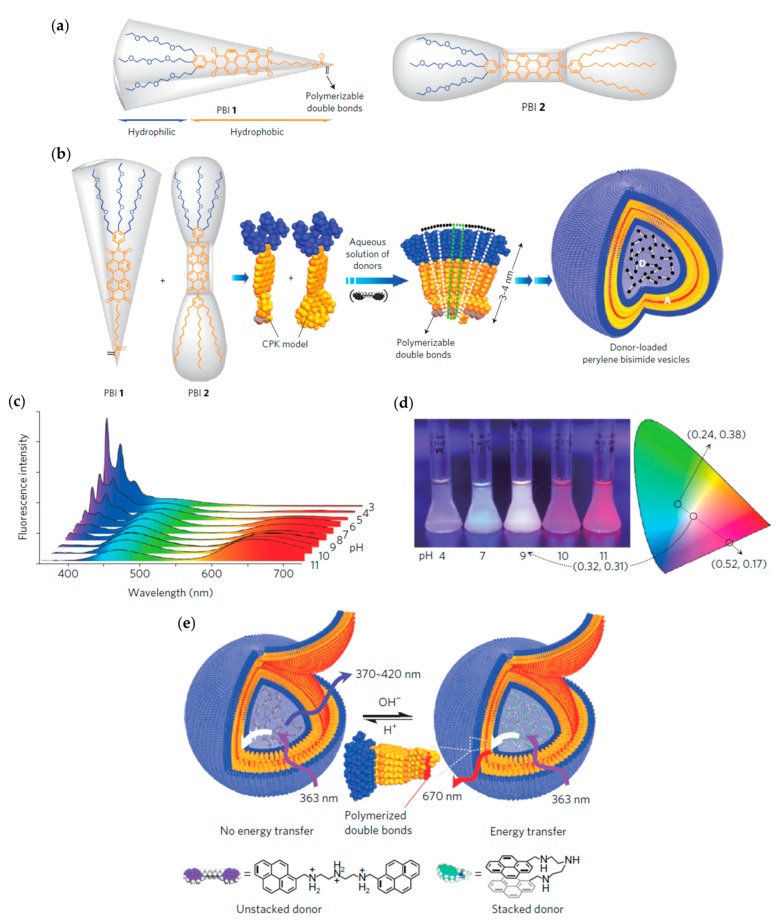
(**a**) Chemical structure of wegde-shaped PBI 1 and dumbbell-shaped PBI 2; (**b**) Schematic illustration based on molecular space-filling (Corey-Pauling-Koltun (CPK)) models for the formation of donor (D)-loaded perylene vesicles in aqueous solution. The water-soluble donor molecules are confined in the aqueous core and the bilayer membrane is composed of perylene bisimide acceptor molecules (A); (**c**) Fluorescence spectra of donor-loaded polymerized vesicles in aqueous solution at pH 3.0–11.0; (**d**) Photograph of donor-loaded polymerized vesicles in aqueous solution at different pH under an ultraviolet lamp (366 nm); (**e**) Schematic illustration of the donor-loaded polymerized vesicles with pH-tunable energy transfer. Copyright Nature, reprinted from Nature Chemistry 2009 [62].

Supramolecular nanostructures with unique photofunctional properties can be made of amphiphiles comprising a fluorescent dye. In the case of high sensitivity of the dye to environmental changes, these nanostructures can be used as fluorescent sensors. Perylene bisimides are fluorescent dyes possessing chemical, thermal and photochemical stability as well as high fluorescence quantum yields [139,140]. Their application ranges from photovoltaic cells to laser dyes and sensors [141,142,143,144,145]. Zhang et al. have synthesized various dumbbell- and wedge-shaped amphiphilic perylene bisimides (PBI) containing a perylene bisimide core and hydrophilic and hydrophobic parts of different size and shapes [61,62,146]. As a function of the molecular shape of the amphiphiles, various morphologies were formed in water. Interestingly, mixing of a dumbbell- (PBI 2) and a wedge-shaped PBI (PBI 1) favored vesicle formation (Figure 13b). An increase of the amount of the dumbbell-shaped amphiphile PBI 2 from (PBI 1)/(PBI 2) = 8/1 to (PBI 1)/(PBI 2) = 4/1 yielded bigger vesicles (from 94 nm to 133 nm). This size change was ascribed to a more demanding volume of the hydrophobic parts leading to a lower curvature and thereby an increase in size. The vesicles were later loaded with hydrophilic bispyrene-based energy donor molecules and displayed fluorescence color changes as a function of the pH of the aqueous environment. This property originated from pH-dependent conformational transformations of the bispyrene donor molecules (Figure 13). Interestingly, at a pH of 9.0 white fluorescence was observed [147]. Because of their pH-sensitive fluorescence emission features, these vesicles might serve as probes for local optical sensing of pH values even within cells or subcellular organelles, thus enabling new insights into physiological processes.

#### 4.1.2. Dendrimersomes as Nanocarriers

##### Drug Delivery: Model Drugs and Anticancer Drugs

Vesicular structures as drug delivery systems (DDS) might help overcoming the limitations of free drugs, such as poor water-solubility, severe side-effects due to a non-selective biodistribution as well as multi drug resistance [148,149,150,151,152,153,154]. Compared to micelles, vesicles have the advantage to encapsulate both hydrophobic as well as hydrophilic cargos. In addition, the incorporation of responsive units in amphiphilic molecules allows the temporally controlled decomposition of the resulting nanostructures through the use of external stimuli, such as pH, temperature or irradiation depending on the nature of the responsive unit [155,156,157,158,159,160].

One of the most effective stimuli is light, as it enables spatial as well as temporal control. Photoresponsivity in DSs was achieved by Nazemi et al. by inclusion of the photolabile *o*-nitrobenzyl unit as junction between the hydrophobic and hydrophilic blocks as well as by positioning this unit repeatedly along a third generation hydrophobic polyester block (Figure 3q) [63]. UV measurements confirmed the photo-triggered release of the model drugs Nile red and fluorescein. Differences in the release profile of the dyes were attributed to their location in the DSs.

Not only light-sensitive moieties but also acid-sensitive functionalities are very useful for the triggered release of anticancer drugs. Indeed, one can take advantage of the more acidic environment of the tumor tissue compared to healthy tissue for controlled drug release [161]. Percec et al. demonstrated a pH-dependent release behavior for DSs assembled from a twin–twin amphiphilic JD comprising carboxylic esters [21]. At an acidic pH of 5.2 the release of the anticancer drug doxorubicin was faster than at physiological pH of 7.4: while drug release at a pH of 7.4 was less than 20% after 100 h, the same value was reached after only a few minutes at a pH of 5.2. After 50 h, a plateau was reached and around 60% of the drug was released.

Finally, onion-like DSs could be an exciting direction for future work on DDS [65]: their multilayer structure may enable a time-dependent release of the cargo located in the different bilayers or between them. Moreover, as shown for onion-like polymersomes, these structures should exhibit a higher drug loading capacity thanks to the higher number of bilayers [162].

##### Gene Delivery

Small interfering RNA (siRNA) therapeutics have received considerable attention for treating hereditary or acquired diseases, such as viral infections or cancer [163]. By targeting the mRNA expression, siRNA can knock down genes (gene silencing). Despite the unique opportunities offered by this therapeutic approach, hurdles remain to be overcome in the delivery of siRNA to the cytoplasm after intravenous injection, mostly because of the siRNA instability against various enzymes and the many biological barriers to be passed before reaching the cytoplasm. Amongst the various nanoplatforms for siRNA delivery, vesicles such as liposomes and polymersomes are attractive systems [164,165], but DSs might be a promising alternative because of the well-defined dendritic structure and the high density of surface groups (multivalency) of their building blocks. Liu et al. reported an amphiphilic JD consisting of a hydrophilic G3 PAMAM dendron linked to an hydrophobic G1 PAMAM dendron terminated with C18 alkyl chains (Figure 3d) [32]. Interestingly, this JD forms DSs, which are transformed into a micellar complex when interacting with siRNA. With this structural transition, the cationic ammonium groups of the dendrimer are more effectively exposed, thereby maximizing the electrostatically induced stabilization of the anionic siRNA. The transformation was confirmed by DLS and TEM measurements as well as by dissipative particle dynamics (DPD) simulations. The micellar complex was shown to protect siRNA from degradation and effectively mediate cell entry by macropinocytosis. Compared to liposomes and polymersomes, these dendritic-based nanoassemblies were able to deliver three types of siRNA (depending on the cell type), namely the heat shock protein 27 siRNA, the dicer-substrate siRNA and the anti-*tat/rev* dsiRNA, into a wide range of cell types such as human cancer cells (prostate cancer PC-3 cells, breast cancer cells MCF7 and MDA-MB231), suspended human T (CCRF-CEM) and B (Jeko-1) cells as well as highly challenging human primary cells and stem cells, such as human primary peripheral blood mononuclear cells (PBMC-CD4^+^), hematopoietic stem cells (HSC-CD34^+^) and glioblastoma stem cells (GSCs) [32].

#### 4.1.3. Dendrimersomes in Diagnostic Imaging

Magnetic resonance imaging (MRI) and fluorescence imaging are monitoring tools used for medical diagnostic imaging. Both techniques differ in their spatial resolution: while MRI provides high-resolution 3D anatomical or structural information, fluorescence visualizes processes on a cellular level [166]. In order to enhance the image contrast, contrast agents, such as gadolinium (Gd)-based complexes (for MRI) or quantum dots (for fluorescence imaging), can be used. However, their small size resulting in a quick clearance by renal filtration and their non-targeted distribution as well as possible toxicity may represent limitations to their successful use in a clinical context. In response to this limitation, contrast agents have been introduced in nano-sized delivery systems, such as liposomes and polymersomes [167,168,169,170]. Vesicular systems are of particular interest as they can encapsulate the contrast agent non-covalently inside their aqueous core or in the bilayer membrane. Besides, it can be covalently attached to the surface of the vesicles. Jang et al. paved the way for the application of DSs as versatile contrast agents for fluorescence imaging. They demonstrated the self-assembly of JDs (Figure 3p) in water or *n*-hexane into hybrid vesicles encapsulating both hydrophilic and hydrophobic CdSe quantum dots [68]. While the alkyl chain length of the JD was shown to determine the morphology of the resulting nanostructure in *n*-hexane, the encapsulation of the quantum dots did not cause any morphological change. Fluorescence imaging showed that hydrophilic 3-mercaptoundecanoic acid (MUA)-capped CdSe/ZnS quantum dots were located inside the aqueous lumen of the vesicles. Given that quantum dots consist of heavy metal ions that can leach out of the nanostructure, toxicity may result and therefore cytotoxicity tests are mandatory [171]. Unfortunately, the stability and biocompatibility of the vesicles were not investigated.

Filippi et al. reported about the application of DSs as potential vector for MRI imaging agents by encapsulating Gd-complexes either in the aqueous inner lumen or inside the membrane [64,71]. Most interestingly, the construction of highly stable, biocompatible Gadoteridol-loaded DSs (Gd HP-DO3A) revealed their potential as relevant alternatives to liposomes. Amphiphilic JDs differing in their topology (twin–twin vs. single–single) and the branching pattern of the hydrophobic part ((3,4)- vs. (3,5)-substitution of the alkyl chains) were used for DS preparation and compared regarding their relaxivity, water permeability and stability in isotonic buffer, human serum as well as in presence of human serum albumin. The DSs were prepared by the film rehydration method in HEPES using a small amount of the charged lipid DSPE-PEG-COOH for stabilization (i.e., for preventing vesicle aggregation). The presence of PEG chains on the surface of vesicles has already shown to improve their stability by providing a strong repulsion between the bilayers [172]. The contrast agent potency of the DSs was confirmed by relaxometry measurements and revealed only a slightly reduced relaxivity (3.2–3.5 mM^−1^·s^−1^) compared to that of the free Gd-complex (4.6 mM^−1^·s^−1^). In comparison to DSs, liposomes generally show a reduced relaxivity due to a slow water exchange rate across the lipid membrane [173]. Even though polymersomes can show a better mechanical stability than DSs, the use of polymersomes as vector for contrast agents on the other hand necessitates the construction of a porous membrane (i.e., more preparation steps compared to that of DSs), as they are significantly less permeable to water than liposomes for instance [167].

Concerning the DSs stability (i.e., capability to retain Gd-complex), the branching pattern of JDs and their topology were shown to play an important role. Compared to their (3,4)-substituted homologs, DSs with a (3,5)-pattern displayed higher stability, which was attributed to the higher packing density of the bilayer membrane due to an interdigitation of the alkyl chains [77]. On the other hand, (3,5)-single–single DSs were more stable in serum albumin compared to the (3,5)-twin–twin DSs. The difference in stability can be explained by stronger interactions between the twin–twin JDs and the serum proteins having destabilizing effects. This is in contrast to previous studies on the interaction of human blood serum with liposomes differing in their steric stabilization. Mohr et al. showed that, because of a steric stabilizing effect, liposomes with a denser packing and multiple hydroxyl groups interact less with plasma proteins than less sterically stabilized liposomes [174]. 

The blood circulation time of MRI contrast agents is an essential criterion for their clinical use [173,175]. DSs displayed circulation times similar to liposomes (70–80 min), but shorter than the ones of polymersomes (>3.5 h) [64,167]. Both studies show the potential of DSs for diagnostic imaging.

#### 4.1.4. Dendrimersomes as Theranostics

**Figure 14:**
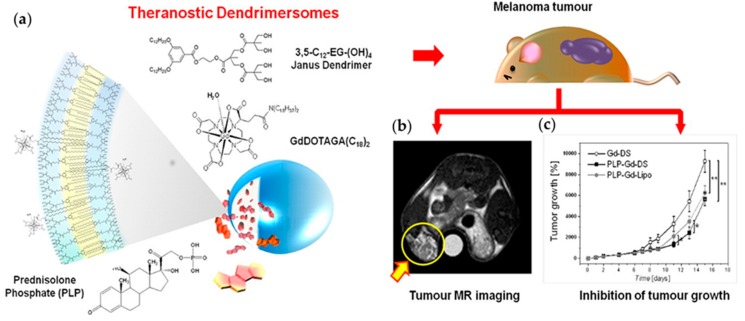
(**a**) Structures of the components of theranostic dendrimersomes: JD 3,5-C_12_-EG-(OH)_4_, the GdDOTAGA(C_18_)_2_ complex (MRI contrast agent incorporated in the membrane bilayer) and the antitumour drug prednisolone phosphate (PLP); (**b**) Representative in vivo MR image showing the tumor after injection of dually-loaded DSs; (**c**) Time course of the tumour growth expressed as the percentage volume increase with respect to the initial tumor volume calculated at time 0 after the systemic administration of the control single-loaded DSs (Gd-DS, white circles), dual-loaded dendrimersomes (PLP-Gd-DS, black squares) or liposomes (PLP-Gd-Lipo, gray circles). Copyright Elsevier, reprinted from Journal of Controlled Release 2017 [81].

Theranostics are macromolecules or nanoparticles that combine both diagnostic and therapeutic applications. Recently, Filippi et al. reported on an in vivo MRI study of theranostic DSs dually-loaded with the anticancer drug prednisolone phosphate (PLP) and a lipophilic Gd-based MRI agent GdDOTAGA(C_18_)_2_ (abbreviated PLP-Gd-DS) (Figure 14a) [81]. In vivo MR imaging performance, therapeutic efficacy and biodistribution of these DSs were determined in mice bearing a melanoma xenograft. PLP-Gd-DSs were also compared to liposomes similarly loaded with PLP and GdDOTAGA(C_18_)_2_ (PLP-Gd-Lipo) that have already been shown to be effective in melanoma treatment [176,177]. PLP-Gd-DSs demonstrated potential for MR imaging as they produced a positive contrast enhancement (T_1_) of ca. 50% in the tumor directly after systemic administration [81]. Within seven days, the contrast decreased to 30.7%. PLP-Gd-DSs were also able to retard the tumor growth up to eight days after administration. Fifteen days after administration, the size of the tumor had decreased of 39.1% (compared to 32.4% for PLP-Gd-Lipo). Studies on the in vivo organ distribution of PLP-Gd-DS revealed maximum localization in the kidney, spleen, and tumor.

#### 4.1.5. Dendrimersomes as Tools for Understanding Lectin Recognition on Cells

**Figure 15:**
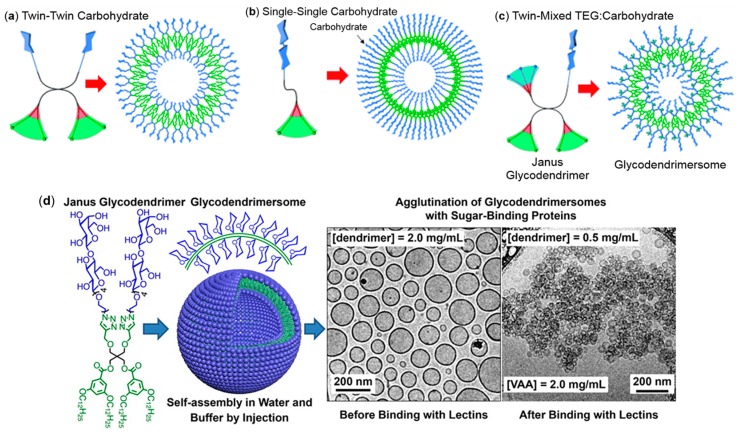
The different topologies of amphiphilic Janus glycodendrimers and the structure of the resulting multivalent GDSs (color code: hydrophilic blue, hydrophobic green, aromatic red) [78]: (**a**) twin–twin carbohydrates; (**b**) single–single carbohydrate; and (**c**) twin–mixed TEG:carbohydrate; (**d**) From left to right: Structure of JGD (3,5)12G1-PE-TRZ-4EOLac2, scheme of its self-assembly as GDSs in aqueous solution, cryo-TEM image of GDSs at JGD concentration of 2 mg/mL, cryo-TEM image of agglutination of GDSs at JGD concentration of 0.5 mg/mL in the presence of toxic mistletoe lectin *Viscum album* agglutinin (VAA) at 2 mg/mL [72]. Copyright Wiley-VCH, adapted from Angewandte Chemie International Edition 2014 [78]; Copyright American Chemical Society, reprinted from Journal of the American Chemical Society 2013 [72].

Most prokaryotic and eukaryotic cells possess a so-called glycocalix on the exterior of the cell, a dense layer of carbohydrates that act as ligands for lectins in cell–cell and cell–matrix interactions. In order to unravel the factors influencing the selective sugar recognition by lectins, several vesicular systems have been used for mimicking the multivalency of biological membranes [178,179,180]. Among those candidates are GDSs prepared by Zhang et al. [72,73,74,78,79,80]. Libraries containing twin–twin, single–single and twin–mixed JGDs (Figure 15a–c, respectively) with d-galactose, d-mannose or d-lactose as hydrophilic part were synthesized and the selective recognition of the resulting GDSs was tested towards various types of plant, bacterial and human lectins [72,73,74,78]. Binding experiments demonstrated that the agglutination efficacy depends on the spatial display of glycan ligands [73,78]. By using JGDs that differ in their topology, a variation of carbohydrate density present at the surface of GDSs was achieved. Twin–mixed JGDs (with two types of hydrophilic groups: carbohydrate and oligoethylene glycol, Figure 15c) were the most efficient in binding lectins compared to their twin–twin (Figure 15a) and single–single (Figure 15b) homologs. Their increased efficacy was ascribed to the reduced steric hindrance between the carbohydrate and lectin. However, the introduction of a spacer in GDSs, which was expected to render the carbohydrate more accessible for lectin binding, led to a decreased agglutination compared to their analogs with a linker [73]. The authors postulated a backfolding of the chains towards the vesicle surface hindering the lectin binding. These results indicate the existence of an optimal balance between carbohydrate density and accessibility. Although the size of the vesicles should influence the number of carbohydrates present on the surface, no specific impact on the bioactivity was observed [73].

Agglutination studies performed with various onion-like GDSs self-assembled from JGDs bearing d-mannose in their hydrophilic part showed a slight dependency on both the position of d-mannose in the JGD and the length of the linker [74].

#### 4.1.6. Dendrimersomes as Tools for Understanding the Role of Membrane Proteins

**Figure 16:**
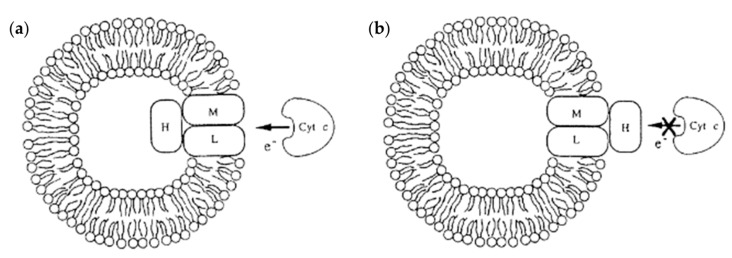
Orientation of RC incorporated in liposomes and determination of preserved functionality. RC reconstituted in vesicles with H-subunit: (**a**) on the inner face, RC can be reduced and is functional; and (**b**) on the outer face, RC cannot be reduced and is not functional. Copyright Japan Society for Bioscience, Biotechnology and Agrochemistry, reprinted from Bioscience, Biotechnology, and Biochemistry 1997 [182].

Because of the essential role of membrane proteins in processes such as cell recognition, energy transduction, signaling and transport phenomena [181]. Considerable attention has been devoted towards a better understanding of their mechanism of action. Investigations at the molecular level in cells are generally challenging because of the complexity of the native membrane. To surmount this hurdle, reconstitution of membrane proteins into simple artificial membrane mimics represents a powerful tool to analyze structural as well as functional aspects. Proteodendrimersomes were produced by Giustini et al. through the incorporation of the photoreaction center (RC)—the integral membrane protein complex of purple bacteria *Rhodobacter sphaeroides—*in DSs [70]. The amphiphilic JD used in this work was a twin–twin JD based on G1 bis-MPA dendron and G1 Percec-type dendron with C12 alkyl chains (Figure 3f). The incorporation of RC in the DSs had an influence on the self-assembling behavior of the amphiphile: in the presence of RC, vesicles with a mean diameter around 300 nm were formed, exhibiting a unimodal size distribution. On the other hand, without RC, the aggregates displayed a trimodal size distribution in the range from 100 nm to 5 µm. Laser-induced absorption experiments confirmed incorporation of RC in a photoactive form. Like many other membrane proteins, RC only shows full activity when its molecular orientation in the bilayer membrane is adequate (H-subunit on the inner face, Figure 16). Charge recombination kinetics proved an almost exclusively vectorial incorporation of RC in proteodendrimersomes (more than 90%), while a comparative study with lecithin vesicles revealed an almost statistical orientation [70]. Experiments with neutral and charged proteoliposomes never reached such a high value and showed that orientation depends on the charge of the lipids [182].

### 4.2. At the Microscale

Because of their cell-like shape and size, giant vesicles are a convenient model to investigate membrane behavior and properties. In contrast to nanometer-sized vesicles, visualization under an optical microscope is possible, facilitating their examination.

#### 4.2.1. Dendrimersomes as Artificial Cells: Integration of Membrane Proteins

Percec et al. used giant DSs for the incorporation of an integral protein—the pore-inducing peptide melittin. DSs containing the fluorophore 1-aminonaphthalene-3,6,8-trisulfonate (ANTS) and the fluorescence quencher α,α’-dipyridinium *p*-xylene dibromide inside their aqueous compartment were prepared via the film rehydration method. The successful incorporation of melittin was demonstrated fluorometrically [21]: the ANTS-fluorescence increased after melittin addition due to the release of the dye through the pores of the integral protein.

**Figure 17:**
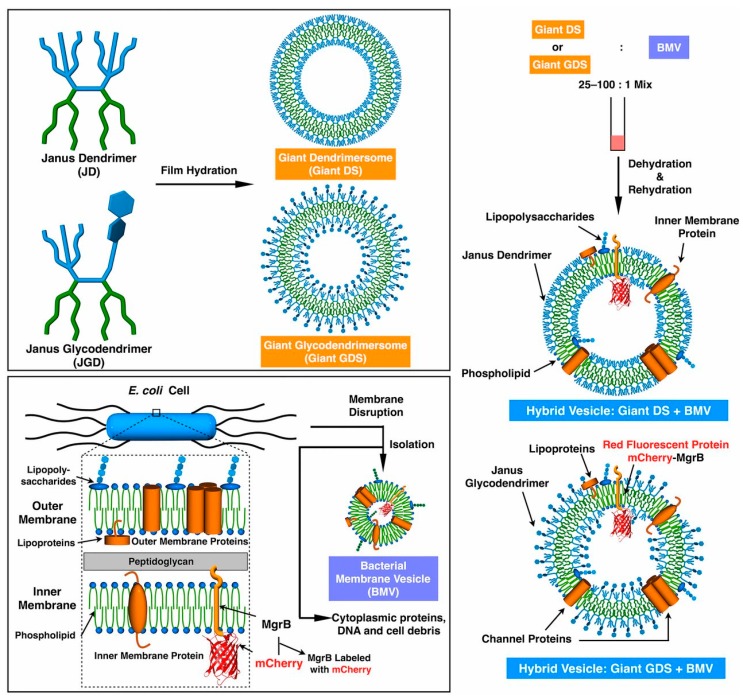
Preparation and coassembly of hybrid giant vesicles from giant DSs, giant GDSs, and *E. coli* BMVs enriched with the transmembrane protein MgrB tagged with the red fluorescent protein mCherry (mCherry–MgrB). Reprinted from [74].

Recently, Xiao et al. published hybrid vesicles prepared by coassembly of amphiphilic JDs or JGDs with bacterial membrane vesicles (BMV) from *E. coli* (Figure 17) [74]. For proof-of-concept, JDs and BMV were labeled with fluorophores and the coassembly was visualized with fluorescence microscopy. Successful incorporation of functional porins into the hybrid vesicles membrane was confirmed by fluorescent dye encapsulation. Such cell-like hybrid vesicles could have great potential as nanomedicines, especially if the coaggregation method can be applied for the preparation of hybrid vesicles with human cells.

#### 4.2.2. Self-Sorting Dendrimersomes

**Figure 18:**
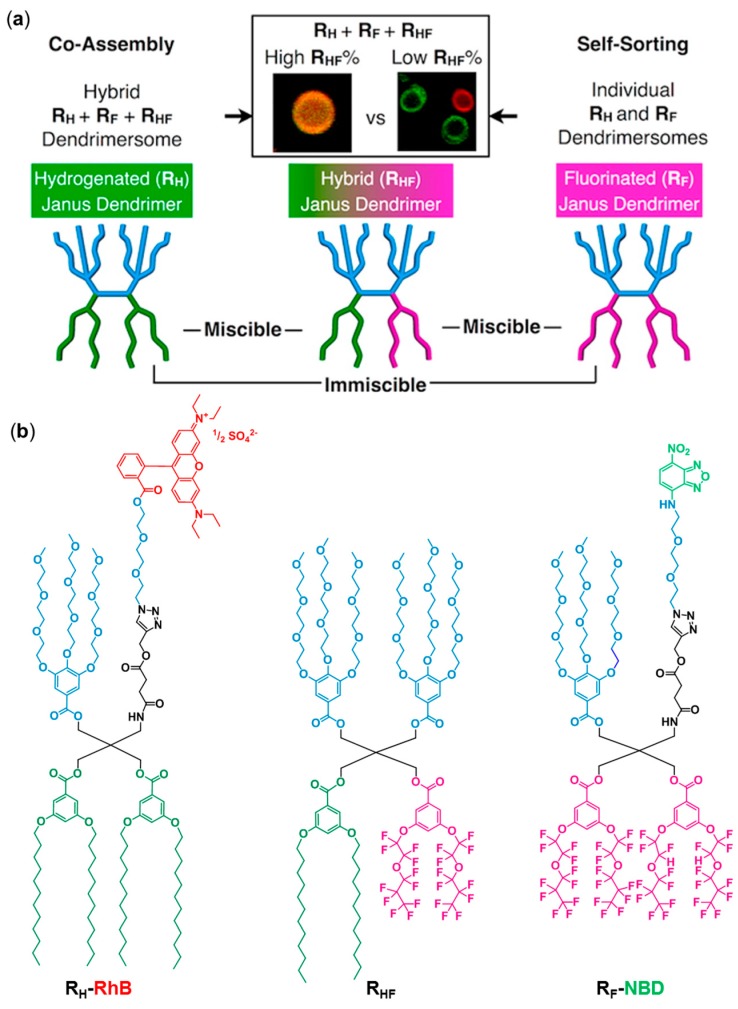
(**a**) Scheme illustrating the coassembly of hybrid R_HF_ Janus dendrimer with both R_F_ and R_H_. Three-component hybrid DSs containing R_H_, R_F_, and R_HF_ were formed when the proportion of R_HF_ was higher than 40%. With low concentration of R_HF_ and in its absence, R_H_ and R_F_ self-sorted into individual R_H_ or R_F_ DSs; (**b**) Structure of the two dye-labeled fluorescent JDs with R_F_ (R_F_-NBD) or R_H_ (R_H_-RhB) chains and non-fluorescent R_HF_. Copyright American Chemical Society, (**a**) reprinted from Journal of the American Chemical Society 2016 [67].

Self-sorting is a phenomenon commonly found in biological systems. It refers to the spontaneous segregation of molecules into discrete complexes within a mixture [183]. A known example of this phenomenon is found in mixed systems that are composed of fluorinated and hydrogenated amphiphiles. Due to the immiscibility of the fluorocarbon and hydrocarbon chains, the surfactants self-assemble into two distinct types of micelles [184,185]. Recently, Xiao et al. have made use of this immiscibility property and investigated the self-sorting ability of hydrogenated (R_H_) (Figure 3j) and fluorinated (R_F_) (Figure 3k) amphiphilic JDs in aqueous media [67]. Fluorescence microscopy demonstrated the preferred coassembly of the dye-labeled JD R_H_-RhB (Rhodamine B) with its complementary unlabeled homolog R_H_, while the dye-labeled JD R_F_-NBD (4-chloro-7-nitrobenzofurazan) (Figure 18b) coassembled with R_F_. On the other hand, mixing of R_F_ with R_H_-RhB and R_H_ with R_F_-NBD led to a phase separation. By using hybrid JDs R_HF_ (Figure 3l and Figure 18b) containing a fluorinated and a hydrogenated dendron, mixing of R_H_ and R_F_ JD was attainable. Furthermore, the coassembly of R_H_, R_F_ and R_HF_ was investigated as a function of their concentration. The phase diagram of the three molecules showed that R_H_ and R_F_ were only completely miscible and coassembled at high R_HF_ concentrations. Mixtures containing ≤34% of R_HF_ with respect to R_H_ and R_F_ resulted in the self-sorting of R_H_ and R_F_ (Figure 18a).

## 5. Conclusions

Amphiphilic Janus dendrimers and dumbbells can be synthesized from the multitude of synthons and synthetic strategies available in organic chemistry. Therefore, they show an unrivaled versatility of building blocks and structures. Vesicles self-assembled from these amphiphilic molecules are a promising alternative to established systems such as liposomes and polymersomes. Their possible applications could range from drug delivery, imaging, and theranostics, to artificial cells. We ought to highlight the important parameters for the vesicles preparation for a rational design of the final structure that could even one day surpass the refinement of the structure they are inspired from: cells.

## Figures and Tables

**Table 1:** Structural features of JDs self-assembling in organic solvents.

Topology	Core	Hydrophobic dendron	Hydrophilic dendron	Solvent	Ref.
Single–single JDs	Ether	G3 Poly(benzyl ether) dendron	Benzyl-terminated aliphatic polyether dendron	Tetrahydrofuran/diisopropylether mixture	[75]
	Ester	G3 Poly(benzyl ether) dendron	G3-dimethylol propionic acid (bis-MPA)	Chloroform	[76]
	Azide	Y-shaped dendron, including two triazoles, central benzene unit and arylalkyl ether with C5 alkyl chains	Aliphatic ether dendron with oligo(ethylene oxide) chains	*n*-Hexane	[68]

**Table 2:** Structural features of JDs self-assembling in aqueous environment: hydrophilic dendrons.

Hydrophilic dendron	Ref.
G1-G3 hydroxy-terminated bis-MPA dendron	[58,74,76]
G2 bis-MPA dendron terminated with tri(ethylene glycol) monomethyl ether chains	[21]
(3,4)/(3,5)/(3,4,5)G1 Percec-type dendron with tri(ethylene glycol) monomethyl ether chains	[21,63,65,66,77]
(3,4)/(3,5)/(3,4,5)G1 Percec-type dendron with tri(ethylene glycol) chains	[21,74]
(3,4,5)G1 Percec-type dendron with glycerol	[21]
G1-G3 thioglycerol dendron	[21]
Saccharide, such as d-mannose, d-galactose, d-lactose	[72,73,74,78,79,80]
G1 Percec-type dendron substituted with tri(ethylene glycol) monomethyl ether chains and saccharides	[73,74,78,79,80]
G1.5/G3 PAMAM-dendron	[32,69]
(3,4,5)G1 Percec-type dendron with quaternary ammonium salts end groups	[21]
(3,4,5)G1 Percec-type dendron with *t*-butylcarbamate end groups	[21]
Aliphatic ether dendron with oligo(ethylene oxide) chains	[68]

**Table 3:** Structural features of JDs self-assembling in aqueous environment: hydrophobic dendrons.

Hydrophobic dendron	Ref.
(3,4)/(3,5)/(3,4,5)G1 Percec-type dendron with C6-C8/C12 alkyl chains or with fluorinated chains	[21,64,65,66,70,71,72,73,74,77,78,79,80,81]
(3,5)G1 Percec-type dendron with C9-C11 alkyl chains	[21]
(3,4,5)G1 Percec-type dendron with C4 alkyl chains	[21]
(3,4)G1 Percec-type dendron with branched hexyl groups	[21,72,78]
G1 bis-MPA dendron terminated with (3,5)-Percec-type dendron with C12 alkyl chains	[73,74]
G2 bis-MPA dendron terminated with C12 alkyl chains	[21]
G3 bis-MPA dendron terminated with C17 alkyl chains	[58]
Arylether dendron	[69,76]
PAMAM dendron terminated with C18 alkyl chains	[32]
Methacryloyl-terminated C6 alkyl chain	[61,62]
(3,4,5)G1 aniline dendron with C12 alkyl chains	[21]
Y-shaped dendron, including two triazoles, central benzene unit and arylalkyl ether with C5 alkyl chains	[68]
Photodegradable G3-polyester dendron with *o*-nitrobenzyl units	[63]

**Table 4:** Structural features of JDs self-assembling in aqueous environment: topology and core.

Topology	Core	Remarks	Ref.
Twin–twin JD	Pentaerythritol	-	[21,64,67,70,71,72,73,74,77,78,79,80]
	Pentaerythritol monosubstituted with fluorescent dye coumarin	To demonstrate coassembly of DSs with bacterial membrane vesicles (BMVs)	[74]
Single–single JD	Amino acids, such as l-alanine, l-phenylalanine, glycine, l-isoleucine, l-valine	For the construction of constitutional isomeric libraries	[65,66]
		H-bonding-motif: formation of onion-like DSs	
	1,2-Propanediol	For the construction of constitutional isomeric libraries	[66]
	Ethylene glycol core	Vesicles for MR-molecular imaging	[64,81]
	Amine/amide-/ester-/ether-/azide-core	-	[21,32,58,65,68,69,73,74,75,76,78,79,80]
	Perylene bisimide	Perylene vesicles as fluorescent pH sensor	[61,62]
